# Health without Medicine

**Published:** 1889-09

**Authors:** Theodore H. Mead


### HEALTH WITHOUT MEDICINE.

BY THEODORE H. MEAD.

No. 3.

In my second article, I have stated what I usually go through with from beginning to end twice a day, taking in the evening the hour before dinner. It has been arranged with special reference to the advice of my good English doctor so as to exercise particularly the upper part of the body, and practiced in a manner to send the blood to the brain. An active young fellow would go through it in fifteen minutes, perhaps ten; it takes me at least twenty and often more. At the same time the excellent counsel to live as much as possible out of doors has not been forgotten, though not closely followed. While still in England I began by renouncing cabs and going everywhere on my own feet, as it presently appeared, much more agreeably and with very little loss of time. In this country the climate does not lend itself in the same degree to out door exercises, but I find no difficulty in walking and riding on horseback with pleasure and profit in all but the very hottest days of summer and I see no reason to doubt that the same would be the case with the great majority of my fellow countrymen and countrywomen.

To return to our in-door exercises, it is evident that others might with equal advantage be chosen by persons of a different physical conformation, and a good book to consult on the subject is the simple and practical little work, "How to Get Strong," by William Blaikie. Whatever the selection may be however, a few points must receive the greatest attention.

In the first place a beginning must be made with great care and moderation particularly by persons in delicate health. The number of repetitions of each exercise given in my list is that which seems to suit me best, but this is a point which each one must settle for himself. The best costume is a suit of wool—a bathing dress for instance, and, until hardened to the exposure, it is well between the exercises to put on a light dressing gown in which to walk about and to recover breath. When you have finished, strip and rub the skin dry and warm with a rough towel.

Let the exercises be sufficiently severe to flush the face and start the perspiration, and between them do not throw yourself into an easy chair, but walk up and down with head erect and shoulders thrown back, drawing long and deep inspirations. While exercising stand straight with head high and lungs fully distended. In this way besides the advantage of the increased supply to the blood of oxygen, the best of tonics, the new acquisitions of muscle will be adapted to keep the chest expanded instead of holding it contracted and compressed as would otherwise be the case.

In forbidding the use of medicine, my English doctor was willing to make but one exception and that was in favor of a little calisaya bark and nux vomica, to be used occasionally, but only occasionlly, as a tonic; and I may here say that on this point, as on every other, the wisdom of his advice—which cost me, if my memory is correct, just three pounds three shillings—has been justified in my case in every particular. We are most of us inclined to attribute to medicine an influence almost magical upon the human system, which a little reflection would show us is quite beyond its powers, and I must confess that for one I should be really ashamed to write down here a list of the drugs of all sorts which I have myself taken, on what might certainly be considered the best advice to be had in this country. Unquestionably they did me, as they have doubtless done most other people more harm than good.

With regard to stimulants of all kinds, my good doctor again made but one exception and this time in favor of the English national beverage, *tea*, to be used morning and evening, but with great moderation. As for all the rest, I had used them always spairngly, and it cost me no effort or regret to give them up, with one notable reservation—how in the world was I to make a breakfast without my coffee? It certainly was dry work for me at first, and many times, if I had given way to my impulse, the tea pot would have taken flight out of the window, for the very sight of it in the morning used to irritate me; but repeated experiments—for this point was not yielded with a stuggle—have convinced me, that coffee predisposes to neuralgic headaches, even if it does not actually bring them on.

The Cold Sponge Bath, at first somewhat dreaded, soon came to be considered a luxury and for many a day has taken rank with me among the necessities of life. To what extent this is the case among our cousins over the water, those of us can best judge who have seen the batteries of sponge bath tubs (looking, as the children always discover, like immense tin hats) waiting to be sent into the bed-rooms of English hotels. In fact the sponge bath has become, we might almost say, a domestic fine art in England. On going to our room at night in an English house, we find the material for it established there as a matter of course and what a picture it brings to our eyes of a sort of domestic service which seems almost too near the ideal ever to be quite realized in our own homes. How swiftly and quietly the tidy, buxom chambermaid brings in the unwieldly vessel; how deftly she places it upon a cloth spread on the floor, and approaches a chair throwing completely over it a large, thick towel while she lays at hand the soap and rough towels for friction; how demurely she asks: "Is there anything else, sir?"

However, these harmless-looking implements or their equivalents must be used by all beginners with care, by persons of delicate health with very great care, for otherwise they are capable of working results directly opposite of those desired. In the first place see to it that the room is warm and free from draughts. Then begin with water scarcely cooler than that you have been in the habit of using for warm baths, and, after sqeezing it copiously over head and shoulders with a big two handed sponge, get out almost as quickly as you got in, and rub briskly from head to foot with a rough towel. By and by you can amuse yourself by polishing up your skin and seeing it grow white and fine, with no more apprehension of ill effects from exposure than have the boys who in summer scramble out of the water and scamper naked along some river bank, but at present get into your clothes without delay. Next day you may lower the temperature of the water, but not more than two degrees, and continue doing so till you have reached the point at which it seems to do you most good, probably somewhere about seventy degrees Fahrenheit. If you do not get a healthy reaction, indicated by a pleasurable glow, or if after a half hour or so your hands or feet get cold, there has been something amiss—either the water has been too cold or you have been too deliberate or the room has not been warm enough. It is well always to put a thermometer into the water, as you cannot judge even approximately by dipping in the hand.

I trust the reader will pardon what may seem to be a lack of good taste in speaking thus at length of my private experience. If my suggestions are of service to any one I shall feel repaid for the effort which it has cost me to do so. It must be evident to every one that if the effects described have been produced in the case of a man already past maturity, much greater may be expected in young persons, perhaps almost in proportion to their youth. In imperfectly developed boys and girls it is probable that by good judgement, watchful care and perserverance, a complete physical transformation may be effected and parents would do well to give the matter timely thought. For them Mr. Blaikie's little book "Sound Bodies for our Boys and Girls" will be found a valuable adviser.

